# Kaposi’s sarcoma overlying Mpox scars – Case report^☆^

**DOI:** 10.1016/j.abd.2025.501186

**Published:** 2025-08-15

**Authors:** José Paulo Ribeiro Júnior, Thaís Barros Felippe Jabour, Liana Franco de Sousa Barros, Juliana Carlos Gonçalves Rego

**Affiliations:** a Department of Dermatology, Hospital Universitário Onofre Lopes, Universidade Federal do Rio Grande do Norte, Natal, RN, Brazil; b Department of Dermatology, Universidade de Santo Amaro, São Paulo, SP, Brazil

Dear Editor,

Kaposi’s Sarcoma (KS) is an angiomatous tumor caused by infection with Human HerpesVirus-8 (HHV-8), often seen in immunocompromised patients, particularly those with HIV/AIDS.^1^ Similarly associated with HIV, there is Mpox, a viral zoonosis with a smallpox resemblance but milder symptoms. It gained global attention during the post-COVID-19 return to normalcy and was declared a Public Health Emergency of International Concern by the World Health Organization in August 2024.^2^ Mpox typically manifests as a flu-like prodrome followed by a smallpox-like rash, primarily after close interpersonal contact.^3^ While KS and Mpox have distinct etiologies and clinical presentations, they share a connection through their unique manifestations in individuals infected with HIV. Currently, there are no published reports describing coexistence, overlap, or interaction between these conditions. We aim to present and discuss a case of KS arising over previous Mpox lesions.

A 22-year-old male patient presented with fever, asthenia, headache, cough, and odynophagia, followed by over 300 umbilicated pustules and crusts on the genital, perianal, and limb regions (Fig. 1A). Mpox was confirmed via PCR from lesion surfaces, and the patient was concurrently diagnosed with HIV. He required hospitalization and treatment with Tecovirimat 600 mg every 12 hours for 14 days. During hospitalization, erythematous-violaceous plaques were observed on the right upper limb, which the patient reported had existed for more than a year (Fig. 1B). Histopathology confirmed the suspicion of KS, configuring AIDS. Around 30 days later, as Mpox lesions progressed into the crusting phase, violaceous plaques appeared on the left thigh over previously healed pustules, progressively enlarging and developing an angiomatous morphology (Fig. 2). A skin biopsy was performed (Fig. 3), with histopathology showing irregular vascular structures with slit-like spaces and proliferation of endothelial cells, along with spindle-shaped cells with nuclei exhibiting both elongated and vesicular patterns. Immunohistochemical analysis demonstrated HHV-8 positivity, along with CD34 and ERG, confirming the diagnosis of Kaposi’s Sarcoma. The patient began chemotherapy with doxorubicin, achieving slight reduction and improvement in violaceous coloration after one year, though residual large anetodermic nodules persisted (Fig. 4). Imaging revealed extension into the quadriceps muscles and iliac/femoral vascular bundles.

**Figure 1:**
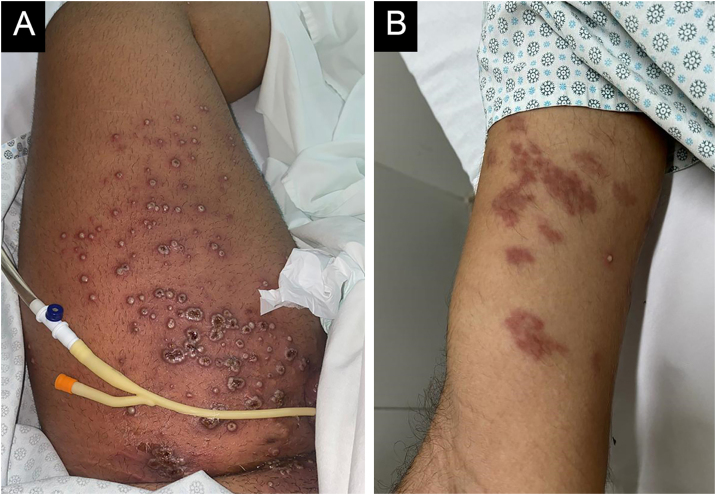
Admission findings of a 22-year-old patient hospitalized for Mpox with a recent HIV diagnosis. Mpox lesions on the patient’s thigh (A) and Kaposi sarcoma lesions present prior to Mpox (B).

**Figure 2:**
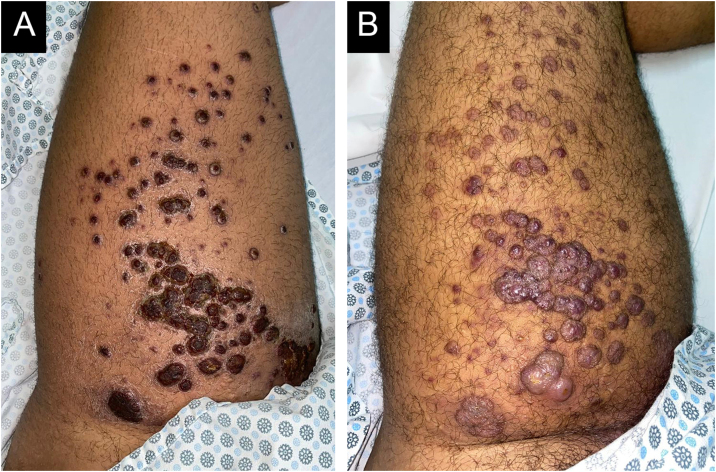
Evolution of lesions from the crusting phase of Mpox (A) into an angiomatous pattern (B), configuring Kaposi sarcoma overlying Mpox scars.

**Figure 3:**
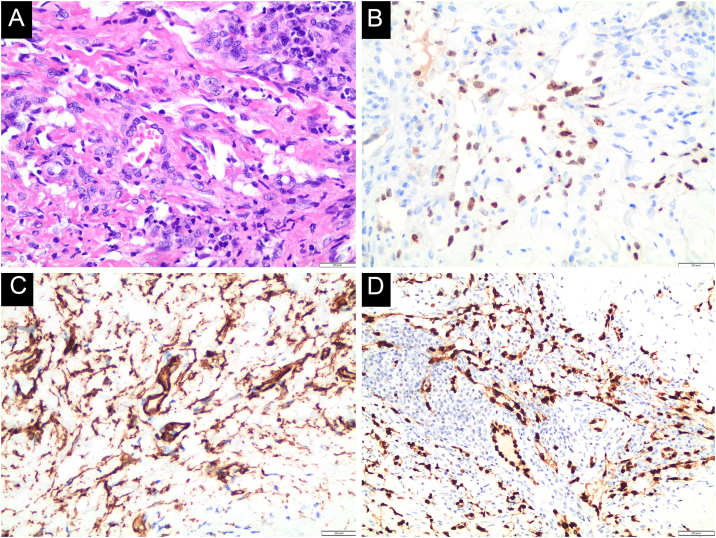
Histopathological findings. A. Hematoxylin & eosin, ×100, showing irregular slit-like vascular spaces lined by endothelial cells and spindle cell proliferation. B. (Immunohistochemical, ×100), positive for Human Herpesvirus type-8 (HHV-8), C. CD34, and D. ETS-Related Gene (ERG).

**Figure 4:**
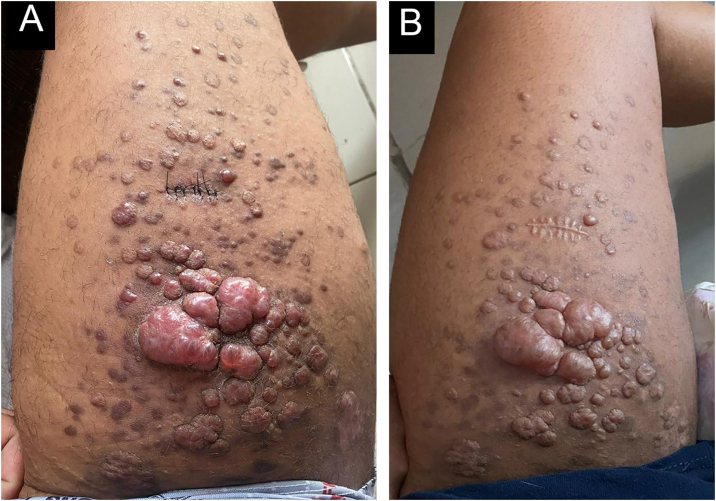
Kaposi sarcoma lesions before (A) and after (B) chemotherapy.

There is a well-known inverse relationship between CD4 count and the risk of KS development.^1^ Immunocompromised individuals, like those with HIV/AIDS, are also at high risk for severe Mpox infection and its sequelae, as seen here with over 300 lesions, qualifying the patient for Tecovirimat treatment.^3^ The development of atypical lesions over the topography of previous Mpox highlights the possible occurrence of phenomena more commonly described in other dermatological conditions. For instance, the Koebner isomorphic response refers to the development of lesions from a pre-existing condition at sites of skin injury, while the Wolf isotopic response describes the occurrence of a new and unrelated disease at the site of a previously resolved disease.^4^, ^5^ A review of over 500 KS patients identified only three cases of the Wolf isotopic phenomenon, all occurring after blistering diseases. None were described after Mpox or other infectious diseases.^6^ Additionally, there are no reports of a Koebner isomorphic response involving Mpox.

In the reported case, KS presented precisely in the configuration of the Mpox lesions, suggesting an isotopic response. However, the patient had pre-existing KS on his arm which appeared before Mpox, complicating classification. Although it is not possible to definitively classify whether the condition represents an isotopic or isomorphic phenomenon, its occurrence can be explained by the old concept of *locus minoris resistentiae*, which describes the susceptibility of a vulnerable or immunocompromised area of the skin to develop lesions.^5^ The Wolf isotopic phenomenon itself has been questioned as a distinct entity by authors such as Happle and Kluger,^5^ who propose that a site previously affected by a dermatosis remains inherently vulnerable. This perspective suggests that all such cases could potentially represent examples of the Koebner phenomenon.

This report introduces a novel presentation, bridging and revisiting concepts typically applied to other dermatoses. It highlights the need to expand diagnostic hypotheses, particularly in light of the increasing prevalence of Mpox cases, and emphasizes early recognition and treatment of this condition.

## Financial support

None declared.

## Authors' contributions

José Paulo Ribeiro Júnior: Design and planning of the study; collection, analysis and interpretation of data; critical review of the literature; drafting and editing of the manuscript or critical review of important intellectual content.

Thaís Barros Felippe Jabour: Collection, analysis and interpretation of data; Critical review of the literature; drafting and editing of the manuscript or critical review of important intellectual content.

Liana Franco de Sousa Barros: Collection, analysis and interpretation of data; critical review of the literature; drafting and editing of the manuscript or critical review of important intellectual content.

Juliana Carlos Gonçalves Rego: Design and planning of the study; critical review of the content; approval of the final version of the manuscript.

## Research data availability

Does not apply.

## Scientific Associate Editor

Hiram Larangeira de Almeida Jr.

## Conflicts of interest

None declared.
